# Reconstructing the Q_o_ Site of *Plasmodium falciparum bc*_1_ Complex in the Yeast Enzyme

**DOI:** 10.1371/journal.pone.0071726

**Published:** 2013-08-12

**Authors:** Cindy Vallières, Nicholas Fisher, Brigitte Meunier

**Affiliations:** 1 Centre de Génétique Moléculaire, Centre National de la Recherche Scientifique, Gif-sur-Yvette, France; 2 Plant Research Laboratory, Michigan State University, East Lansing, Michigan, United States of America; University of Melbourne, Australia

## Abstract

The *bc*_1_ complex of the mitochondrial respiratory chain is essential for *Plasmodium falciparum* proliferation, the causative agent of human malaria. Therefore, this enzyme is an attractive target for antimalarials. However, biochemical investigations of the parasite enzyme needed for the study of new drugs are challenging. In order to facilitate the study of new compounds targeting the enzyme, we are modifying the inhibitor binding sites of the yeast *Saccharomyces cerevisiae* to generate a complex that mimics the *P. falciparum* enzyme. In this study we focused on its Q_o_ pocket, the site of atovaquone binding which is a leading antimalarial drug used in treatment and causal prophylaxis. We constructed and studied a series of mutants with modified Q_o_ sites where yeast residues have been replaced by *P. falciparum* equivalents, or, for comparison, by human equivalents. Mitochondria were prepared from the yeast *Plasmodium*-like and human-like Q_o_ mutants. We measured the *bc*_1_ complex sensitivity to atovaquone, azoxystrobin, a Q_o_ site targeting fungicide active against *P. falciparum* and RCQ06, a quinolone-derivative inhibitor of *P. falciparum bc*_1_ complex.The data obtained highlighted variations in the Q_o_ site that could explain the differences in inhibitor sensitivity between yeast, plasmodial and human enzymes. We showed that the yeast *Plasmodium*-like Q_o_ mutants could be useful and easy-to-use tools for the study of that class of antimalarials.

## Introduction

Malaria is among the most serious health problems in the world leading to more than 655,000 deaths in 2010 mainly among children under five years old, according to the World Malaria report 2011. Although several antimalarial drugs have been developed, emerging resistance to many of these compounds and dissemination of drug-resistant *P. falciparum* are compromising the treatment of malaria patients. Thus there is an urgent need for new antimalarial drugs. The complex III or *bc*_1_ complex is an attractive target for antimalarial drugs. This respiratory chain complex is essential for *P. falciparum* proliferation as its catalytic activity is critical for the maintenance of the mitochondrial membrane potential and for the reoxidation of ubiquinol, which is needed for the ubiquinone-dependent dihydroorotate dehydrogenase, and by consequence for the biosynthesis of pyrimidine (see for instance [1]). In addition, differences in the sequences of the active sites of the *bc*_1_ complex between organisms facilitate the search for inhibitors with selective activity.

The hydroxynaphthoquinone atovaquone is currently the sole *bc*_1_ complex inhibitor used (in combination with proguanil as Malarone®) as antimalarial drug in treatment and causal prophylaxis. The compound is also used to treat *Pneumocystis jirovecii* pneumonia, *Toxoplasma gondii* toxoplasmosis, and other infections. In the USA, Malarone® prescription accounted for more than half of all antimalarial prescriptions. However, the cost of atovaquone is so far prohibitive for more general use. The patent for Malarone® expires this year, which might result in lower cost generics. With the possibility of more extensive use of atovaquone, the risk of resistance to atovaquone would likely increase.

*P. falciparum* atovaquone-resistant parasites have been reported to emerge during atovaquone-proguanil therapy, leading to typical treatment failure [2], [3]. The resistance is caused by point mutations in the drug target. Therefore, new drugs that could circumvent the resistance would be required. Different compounds are currently being studied, such as 4(1*H*)-pyridones, acridones, acridinediones, and 4(1*H*)-quinolones (see for instance, [4] and also [5], [6]). Known drugs used to control animal parasites [7] or plant pathogenic fungi [8] have been also revisited. It has been reported, for instance, that the fungicide azoxystrobin that targets the *bc*_1_ complex was a potent inhibitor of *P. falciparum* [8].

The *bc*_1_ complex contains two sites that bind its substrate quinol and inhibitors, the Q_o_ and Q_i_ sites. Atovaquone targets specifically the Q_o_ site. The Q_o_ site (and the Q_i_ site) is provided by the main subunit of the *bc*_1_ complex, cytochrome *b*. Cytochrome *b* is mitochondrially encoded in all eukaryotes while the other subunits of the complex are nuclearly encoded. The Q_o_ site is a relatively large domain formed from components encompassing amino acid residues 120–150 and 260–280 of the cytochrome *b*. Hydrogen bonds form a ‘loose stitching’ that allows a degree of expansion of the site on occupant binding [9]. Some sidechains within the Q_o_ site, such as components of the ‘PEWY’ motif (residues 271–274, yeast numbering), demonstrate considerable conformational flexibility on inhibitor binding [10]. The Q_o_ site forms a bifurcated volume. On the basis of structural and mutational analysis, Q_o_ inhibitors can be separated into two classes by their mode of binding [9], [10]. For instance, azoxystrobin binds in the so-called ‘haem *b*_l_ proximal’ domain of the Q_o_ site whereas stigmatellin binds in the ‘haem *b*_l_ distal’ domain, occupying the region of the Q_o_ site close to the interface with another catalytic subunit of the complex, the iron-sulphur protein (ISP). Crystal structures for several inhibitor-liganded *bc*_1_ complexes are available, such as azoxystrobin- and stigmatellin inhibited enzymes (see for instance, [10]–[12]). A crystal structure for the atovaquone-inhibited complex is not yet available. However, atovaquone is likely to be a ‘distal’ binding inhibitor due to its effect on the EPR spectrum of the ISP [13]. The quinolone compound RQC06 that showed a promising antimalarial activity might also bind in the ‘distal’ region of the Q_o_ pocket, as suggested by the modelling analysis [14].

In order to facilitate the study of drugs targeting the *P. falciparum bc*_1_ complex, we are developing the yeast (*Saccharomyces cerevisiae*) model. By modifying the inhibitor binding sites of the yeast native *bc*_1_ complex, new versions can be generated that mimic the *P. falciparum* enzyme. In this study, we constructed a series of yeast mutants harbouring variants of the Q_o_ site where yeast residues have been replaced by *P. falciparum* equivalents, or, for comparison, by the human equivalents. The yeast mutants with *Plasmodium*-like (and human-like) Q_o_ site were used to investigate the structural determinant of the differential sensitivity to atovaquone and azoxystrobin and to explore the binding mode of the novel molecule RCQ06. We also constructed a mutant harbouring the atovaquone resistance mutation Y279S (Y268S in *P. falciparum* sequence) in the *Plasmodium*-like enzyme to study the effect of this mutation on new inhibitors such as RCQ06. The yeast mutants could be useful tools for the study of new drugs with selective activity and for the analysis of resistance mutations.

## Materials and Methods

### Materials

Equine cytochrome *c*, decylubiquinone, azoxystrobin and atovaquone were obtained from Sigma. RCQ06 was kindly supplied by Prof. Paul O’Neill (Liverpool, UK).

### Yeast Mutants

The mutants were generated by side-directed mutagenesis and mitochondrial transformation as described in [15]. They have identical nuclear and mitochondrial genomes with the exception of the mutations introduced in the cytochrome *b* gene.

### Measurement of Quinol:Cytochrome *c* Reductase Activity

Yeast mitochondria were prepared as in [16]. Bovine mitochondrial samples were kindly given by Prof. Peter Rich (UCL, UK). Quinol:cytochrome *c* reductase activity measurements were performed in 10****mM potassium phosphate pH 7 and 20µM equine cytochrome *c* at room temperature. Mitochondria were diluted to 5–30 nM *bc*_1_ complex. Concentrations of monomeric *bc*_1_ complex were determined from the cytochrome *b* α-band in dithionite-reduced optical spectra, using ε = 28.5 mM^−1^.cm^−1^ at 562 nm *minus* 575 nm. Activity was initiated by the addition of 20µM decylubiquinol. Cytochrome *c* reduction was recorded at 550 nm *versus* 540 nm over a 3-min time-course in a Beckmann DU 640 spectrophotometer. Initial rates were measured. From these rates, turnover numbers (TN) were calculated as cytochrome *c* reduced per *bc*_1_ complex per second.

### Inhibitor Titration

Cytochrome *c* reduction activity was measured as described above in presence of increasing concentrations of inhibitors (six to ten different concentrations). Each measurement was repeated at least twice and averaged. The errors did not exceed 10%. The mid-point inhibition concentrations (IC_50_) were determined from the titrations. As the titrations were performed using mitochondrial samples containing different concentrations of *bc*_1_ complex (5–30 nM), the results were presented as ratio of IC_50_ on the concentration of *bc*_1_ complex.

### Ligand Docking and Molecular Modelling

Atovaquone was docked into the Q_o_ site of yeast cytochrome *b* (3CX5.PDB) as described in [17]. An atomic model of RCQ06 was created using PRODRG2 and the Dock Prep module of Chimera [18], [19]. The energy-minimised RCQ06 model was docked into a 9Å radius sphere centred on the ε2-oxygen atom of cytochrome *b* residue E272 in the Q_o_ site of 3CX5.PDB using EADock DSS *via* SWISSDOCK [20]. Using an iterative search, ligand binding modes with favourable CHARMM energies were clustered taking account of the solvent effect with the FACTS implicit solvation model, and the resulting output files examined with Chimera and VMD.

## Results and Discussion

### 1) Sensitivity to Q_o_ Inhibitors: Comparison between Plasmodial, Mammalian and Yeast *bc*_1_ Complexes

The sensitivity of the *bc*_1_ complex (cytochrome *c* reductase) activity towards atovaquone, azoxystrobin and RCQ06 is presented in Table 1. As previously reported, yeast *bc*_1_ complex, as *P. falciparum* enzyme, is highly sensitive to atovaquone (IC_50_ of 4 (molar ratio)) while the bovine enzyme was less reactive (IC_50_ of 75). Azoxystrobin has been shown to be a potent inhibitor of *P. falciparum* proliferation with an IC_50_ in the nanomolar range (15 nM [8]). The drug inhibits yeast *bc*_1_ complex with an IC_50_ of 20 while it is less active against the bovine enzyme (IC_50_ of 180). RCQ06 was also reported as potent inhibitor of the plasmodial *bc*_1_ complex [14] with an IC_50_ of around 1.3 nM, which is in the same range than the atovaquone IC_50_ (around 3 nM) [21]. We found that the bovine enzyme was less sensitive to that compound (IC_50_ of 40). The differential sensitivities to RCQ06 and atovaquone between the *Plasmodium* and mammalian enzymes are thus similar. By contrast, the yeast enzyme was highly resistance to the RCQ06 (IC_50_>500).

**Table 1:** *bc*_1_ complex activity and sensitivity to Q_o_ site inhibitors.

*bc*_1_ complex source	TN (%)	IC_50_		
		atovaquone	azoxystrobin	RCQ06
*P. falciparum*	nd	3 nM	nd	1.3 nM
bovine	nd	75	180	40
Yeast WT	100	4	20	>500
*Plasmodium*-like mutants				
PF1	52	2	5	>500
PF2	105	10	16	10
PF8	107	16	220	40
PF11	44	3	40	25
PF12	15	>850	30	>500
Y279S	27	>850	60	>500
Human-like mutants				
L275F	110	150	30	10
F278A	61	35	120	–
M295L	120	10	45	–
HSa	81	200	130	–
HSb	47	170	160	–
HSc	89	60	140	–

**Figure 1:**
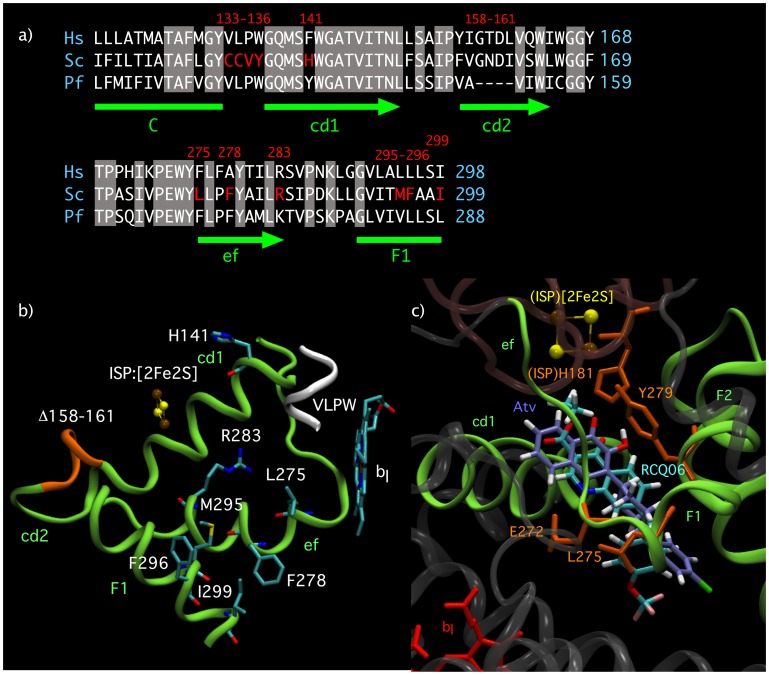
Sequence and structure of the Q_o_ site. (A) Comparison of cytochrome *b* sequence. Regions of the polypeptide forming the Q_o_ domain and its vicinity are shown. The Q_o_ site itself is formed by residues located in the regions 120–150 and 260–280. Mutated residues studied here are highlighted in colour. Green arrows and bars indicate structural components, as shown in panels B and C: C and F1 are transmembrane helices; cd1 and cd2, extramembrane short helices; ef, a loop containing highly conserved residues. Hs, human; Sc, yeast; Pf, *Plasmodium falciparum*. (B) Location of the mutated residues in the yeast *bc*_1_ Q_o_ site. The figure was drawn using the coordinates of 3CX5.PDB [39]. VLPW, location of the mutation CCVY_133–136_VLPW. (C) Molecular model of atovaquone (lilac CPK) and RCQ06 (cyan CPK) docked in the Q_o_ site of yeast cytochrome *b* (3CX5.PDB [39]). Selected sidechains from cytochrome *b* and the ISP are represented in orange. The alpha carbon backbones of cytochrome *b* and the ISP are represented in cartoon form in grey/green and dark pink respectively. Helices of interest within cytochrome *b* forming structural elements of Q_o_ are labelled in green. Also shown are the haem *b*_l_ and [2Fe2S] prosthetic groups of cytochrome *b* and the ISP. Residue notation corresponds to the yeast enzyme. Atv, atovaquone.

The mutated residues in the yeast strains are presented in Table 2 for the *Plasmodium*-like mutants (PF) and in Table 3 for the human-like mutants (HS). Their location in the sequence and the structure is shown in Fig. 1.

TN, turnover number: cytochrome *c* reduced per *bc*_1_ complex per second (Materials and Methods). The values are presented as % of the WT activity (140 s^−1^).

IC_50_, mid-point inhibition concentration.The IC_50_ values for bovine and yeast enzymes were obtained as described in Materials and Methods. The values are presented as ratio of IC_50_ on the concentration of monomeric *bc*_1_ complex (estimated using cytochrome optical signal as in Materials and Methods). For example, 4 molecules of atovaquone were added per yeast WT monomeric *bc*_1_ complex to inhibit the quinol cytochrome *c* reductase activity by 50%.

For *P. falciparum* enzyme, the atovaquone IC_50_ is taken from [21]. In the same study, the IC_50_s for bovine and human enzymes were approximately 70 nM. The RCQ06 IC_50_value is from [14]. Note that the *bc*_1_ complex concentrations in the *P. falciparum* preparations used for inhibitor titrations were not available in the published studies. It was thus not possible to present the data as the molar ratio IC_50_/[*bc*_1_ complex]. An IC_50_ of azoxystrobin is not available. The inhibitor was reported to be highly active on the parasite growth [8].

In order to study the structural determinants that could explain the differential sensitivity to these drugs, we monitored the impact of mutations introduced in the Q_o_ site of the yeast *bc*_1_ complex, single mutations and combined *Plasmodium*- or human-like changes.

### 2) Determinants of Atovaquone Sensitivity Studied in *Plasmodium*-like Yeast Q_o_ Site Mutants

In previous works, the low atovaquone sensitivity of the bovine *bc*_1_ complex (as compared to the sensitivity of the yeast enzyme) was investigated. A computed energy-minimised structure for atovaquone liganded to the yeast *bc*_1_ complex suggested that residue 275 plays a key role in the differential sensitivity. The presence of a phenyalanine at position 275 in the bovine enzyme would hinder the drug binding. F275 being replaced by L in the yeast enzyme, the steric constraint is lessened and the sensitivity to atovaquone increases. When the mutation L275F was genetically introduced in the yeast *bc*_1_ complex, its sensitivity was significantly decreased (over 20-fold) [13], [15], [22].

In the cytochrome *b* of *P. falciparum* (and of other *Plasmodium* and of *Toxoplasma*), the F275 variant is naturally present. However the parasite *bc*_1_ complex is highly sensitive to atovaquone. Thus other structural variations should explain the differential sensitivity between the parasite and the mammalian enzymes.

Comparison of the cytochrome *b* sequences (Figure 1A) shows that the Q_o_ domain is well conserved between organisms. There are however variations that may affect atovaquone susceptibility.

In order to investigate their impact, we constructed a series of yeast strains harbouring multiple changes in the Q_o_ domain. We replaced, in a step-wise manner, ten yeast residues by the *P. falciparum* equivalents (see Table 2): in positions 133–136 and 141 (mutant PF1), positions 133–136, 141 and 275 (mutant PF2), positions 275 to 299 (mutant PF8) and finally a combination of the ten changes from residue 133 to residue 299 (mutant PF11). The location of the mutated residues in the structure is shown in Figure 1B. The *bc*_1_ complex level was not affected in the mutants produced in this study, as judged by the optical signal of cytochromes (not shown). None of the modifications introduced in the Q_o_ site severely affected the overall assembly of the complex.

**Table 2:** *Plasmodium*-like mutations in the yeast Q_o_ site.

strains	mutated residues and positions										
WT	C133	C134	V135	Y136	H141	L275	Y279	R283	M295	F296	I299
L275F	–	–	–	–	–	F	–	–	–	–	–
PF1	V	L	P	W	Y	–	–	–	–	–	–
PF2	V	L	P	W	Y	F	–	–	–	–	–
PF8	–	–	–	–	–	F	–	K	V	L	L
PF11	V	L	P	W	Y	F	–	K	V	L	L
PF12	V	L	P	W	Y	F	S	K	V	L	L
Y279S	–	–	–	–	–	–	S	–	–	–	–

These residues are shown in the sequence comparison (Figure 1A). Their location in the Q_o_ structure is presented in Figure 1B. –, unchanged residue.

In addition to substitutions in the Q_o_ site, the sequence comparison (Figure 1A) showed a deletion of four amino acids, Δ158–161, in *P. falciparum* cytochrome *b*. The deletion, observed in all apicomplexan, is located in helix cd2 in the vicinity of the Q_o_ site (Figure 1B). The deletion, introduced in PF11, caused a significant decrease in *bc*_1_ complex activity but had little effect on atovaquone sensitivity (not shown). The study of the deletion and of its possible compensations will be reported elsewhere.

It should be noted that the acquired atovaquone-resistance mutations found after drug exposure of parasites (*Plasmodium* sp., *T. gondii* and *P. jirovecii*) are located at positions 139, 147, 148, 150, 152, 266, 269, 275, 278, 279, 282, 283, 286, 291 and 295 [23]–[29]. Most residues are conserved in yeast and *P. falciparum*, with the exception of residues 275, 283 and 295. Thus the *Plasmodium*-like mutations studied here are located at residues or closed to residues involved in acquired-atovaquone resistance.

The Q_o_ pocket of PF11 best mimics *P.falciparum* Q_o_ site as it harbours most of the variations that might affect the susceptibility to atovaquone or other Q_o_ site inhibitors. The combined change was well tolerated: the *bc*_1_ complex retained more than 40% activity (Table 1).

Interestingly, PF11 was highly sensitive to atovaquone despite the presence of F275 (IC_50_ of 3) whereas F275 alone in the yeast Q_o_ site (mutant L275F) caused a strong atovaquone resistance (IC_50_ of 150). A similar behaviour was observed with PF2 and PF8 but the atovaquone sensitivity was less marked (IC_50_ of 10 and 16, respectively). In *Rhodabacter capsulatus* cytochrome *b*, a phenylalanine is naturally present at this position. The IC_50_ of atovaquone was estimated to be around 30 nM. Interestingly, the sensitivity of the bacterial complex to atovaquone was increased (IC_50_ decreasing from 30 nM to 10 nM) by the introduction of two mutations replacing bacterial residues by the plasmodial equivalents, namely I281M and R283K (yeast numbering) [30], which is in agreement with our data as R283K is present in PF8. It seems therefore that when L275F is combined to other *Plasmodium*-like mutations, the impact of L275F is weakened. From these data, it appears that the atovaquone sensitivity of *P. falciparum bc*_1_ complex could be explained by a combination of variations (variations from the yeast Q_o_ site) that cancel the resistance effect of F275. We could hypothesise that the *P. falciparum* Q_o_ site and the yeast *Plasmodium*-like Q_o_ site have a larger volume that could accommodate atovaquone despite a phenylalanine in position 275. The modified yeast *bc*_1_ complex harbouring a *Plasmodium*-like Q_o_ and highly susceptible to atovaquone (PF11) could be useful model to study the effect of resistance mutations.

### 3) Determinants of Atovaquone Resistance Studied in Human-like Yeast Q_o_ Site Mutants

The mammalian *bc*_1_ complex is naturally resistant to atovaquone (as compared to the plasmodial and the yeast enzymes) (Table 1).

To investigate further the observed resistance, we modified the Q_o_ site of yeast *bc*_1_ complex and introduced human variations, single or combined (Table 3). The substitutions were well accommodated in yeast Q_o_ site. The mutant enzyme combining eight human changes (HSc) showed nearly WT activity (89%).

**Table 3:** Human-like mutations in the yeast Q_o_ site.

strains	mutated residues and positions							
WT	C133	C134	V135	Y136	H141	L275	F278	M295
L275F	–	–	–	–	–	F	–	–
F278A	–	–	–	–	–	–	A	–
M295L	–	–	–	–	–	–	–	L
HSa	–	–	–	–	–	F	A	–
HSb	–	–	–	–	–	F	A	L
HSc	V	L	P	W	F	F	A	L

These residues are shown in the sequence comparison (Figure 1A). Their location in the Q_o_ structure is presented in Figure 1B. −, unchanged residue.

The three human-like mutations, L275F, F278A and M295L, conferred some level of resistance towards atovaquone. The IC_50_ increased 35-, 9- and 2.5-fold in L275F, F278A and M295L, respectively. The combination of these mutations (HSa and HSb) caused a further increase of resistance. When combined with amino-acid changes at positions 133 to 141 (HSc), the resistance to atovaquone was decreased, as observed with *Plasmodium*-like mutants. However, the human-like *bc*_1_ complex remained 15-fold more resistant than the WT enzyme, with an IC_50_ value of 60, similar to the IC_50_ value of 75 obtained with the bovine enzyme. It can thus be suggested that the combination of F275, A278 and L295 in human *bc*_1_ complex is responsible for the natural resistance towards atovaquone.

### 4) Sensitivity and Resistance to Azoxystrobin of *Plasmodium*- and Human-like Mutants

In the human-like mutants, F278A caused a 6-fold increase in azoxystrobin resistance, M295L and L275F had a weaker effect. The triple mutation (HSb) conferred 8-fold resistance. Changes in position 133 to 141, when combined with that triple mutation (HSc), did not affect the resistance (IC_50_ of 140). Thus the 9-fold resistance of the mammalian enzyme to azoxystrobin (IC_50_ of 180), compared to yeast enzyme (IC_50_ of 20) could be explained by the combination of F275, A278 and L295 in the binding site.

In the *Plasmodium*-like yeast mutants, modifications of residues 283 to 299 caused a 10-fold increase in resistance (IC_50_ of 220 for PF8), but the changes in position 133 to 141 lowered the resistance by 5 fold (IC_50_ of 40 for PF11). That combination of residues might explain the susceptibility of *P. falciparum* to azoxystrobin.

As a control, we compared the sensitivity to the Q_i_ site inhibitor antimycin of the *bc*_1_ complex of the human- and the *Plasmodium*-like mutants, HSc and PF11, with that of the WT control. No or very little difference was observed (data not shown).

### 5) Effect of the Acquired Atovaquone Resistance Mutation Y279S (Y268S in *P. falciparum*)

In *P. falciparum* isolated from patients after treatment failure, three mutations have been reported, Y279S/C/N (Y268 in *P. falciparum*). Mutations Y279S and C are the most frequent mutations in the parasite. Their consequences on the catalytic activity and the stability of the complex have been characterized in the *P. falciparum* (Y268S) [17], the yeast [31], [32] and the bacterial enzyme [30], [33].

We introduced the mutation Y279S in PF11 to obtain a model mimicking *P. falciparum* resistant enzyme. As presented in Table 1, the resulting mutant enzyme (PF12) showed a more severe decrease in activity, retaining only 15% of the WT activity. This was expected as the mutation Y279S alone in the yeast Q_o_ site caused a 70% decrease of the *bc*_1_ complex activity. In the parasite *bc*_1_ complex, the acquired resistance mutation resulted in a low activity and unstable ISP [17]. It has been suggested that residue Y279 is involved in the correct orientation of the quinol bound in the Q_o_ site *via* tyrosyl-benzoquinone hydrophobic packing, facilitating quinol deprotonation and electron transfer to the ISP [34]. Substitution of the tyrosine by (the less bulky) serine is likely to abolish, or otherwise weaken, this predicted stabilising interaction interfering with optimal quinol binding, potentially slowing the reduction of the ISP [2Fe2S] cluster redox group.

When introduced in the *Plasmodium*-like Q_o_ site (PF12) or in the yeast Q_o_ site, Y279S caused a high level of resistance to atovaquone (IC_50_>850), similar to that observed with the Y268S plasmodial enzyme. By contrast, the sensitivity to azoxystrobin was little affected by the atovaquone resistance mutation (IC_50_ of 40 and 30 for PF11 and PF12, respectively). This result was not unexpected since azoxystrobin has been shown to bind in the Q_o_ site at a position proximal to the haem *b*_l_ [10], while atovaquone binds at a position distal to the haem *b*_l_ [13]. However it emphasizes the interest of developing molecules, such as azoxystrobin, that have both differential reactivity and can circumvent the atovaquone-resistance mutation.

### 6) RCQO6 Binding, Comparison with Atovaquone

**Figure 2:**
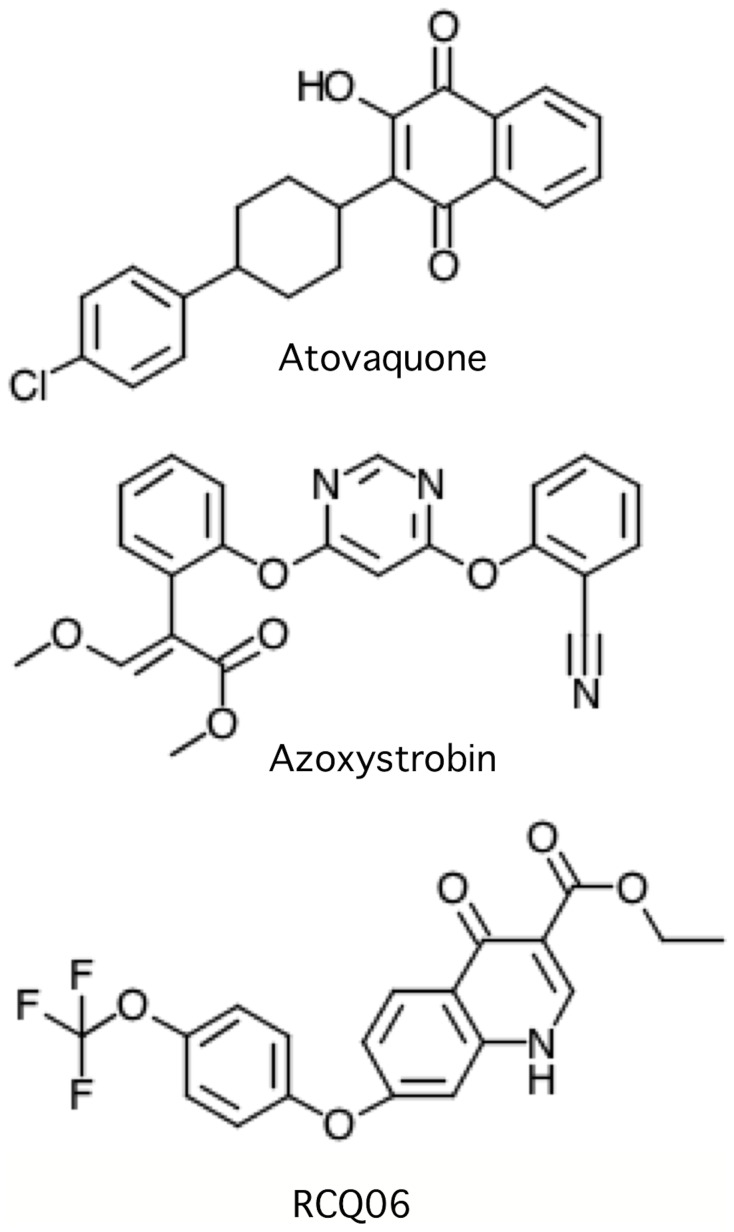
Chemical structures of atovaquone, azoxystrobin and RCQ06.

As shown in Table 1, the yeast *bc*_1_ complex is highly resistant to RCQ06. Strikingly, the introduction of mutation L275F rendered the enzyme over 50-fold more sensitive to RCQ06 than WT. The sensitivity to RCQ06 was observed in all the mutants with L275F in the Q_o_ site: PF2, PF8, and PF11. Thus RCQ06 binding is likely to be stabilised by an aromatic residue at position 275, whereas the binding of atovaquone is hindered by the bulkier residue. The chemical structures of RCQ06, atovaquone and azoxystrobin are presented in Figure 2.

The introduction of the atovaquone resistance mutation Y279S in *Plasmodium*-like Q_o_ site (PF12) caused a high level of resistance to RCQ06, as it did for atovaquone. This suggests that the two inhibitors have similar binding mode. On the basis of this mutational analysis, a model of RCQ06 has been build. Figure 1C presents a molecular model of atovaquone and RCQ06 docked into the Q_o_ site of the yeast *bc*_1_ complex. As mentioned in the introduction, a crystal structure of the atovaquone-bound enzyme is not available but atovaquone is predicted to interact with the Q_o_ site in a manner similar to that of stigmatellin *via* hydrogen bonding from its naphthoquinone moiety *via* a bridging water molecule to E272 (yeast numbering) of cytochrome *b*, with a second hydrogen bond formed to the imidazole group of the ISP [2Fe2S] cluster ligand H181 [13].

Our model suggests that RCQ06 binds in a manner reminiscent to that of stigmatellin with a 3.5Å hydrogen bond between its quinolone amine moiety and the glutamyl sidechain of cytochrome *b* residue E272. This association would require that the glutamyl is acting as the hydrogen bond acceptor, and is thus present in the deprotonated (carboxylate) form, which is thermodynamically accessible when haem *b_l_* is oxidised [35]. In the docking model, the quinolone carbonyl of RCQ06 is within hydrogen bonding distance of [2Fe2S] cluster ligand H181 (2.8Å), although the formation of such a hydrogen bond would require the histidyl sidechain of this residue to be present as a protonated imidazole, favouring the reduced form of the [2Fe2S] cluster. Perhaps surprisingly, we predict no hydrogen bonding interactions between the trifluoromethoxy group of RCQ06 and the surrounding protein. A stabilising aliphatic/aromatic interaction (2.7Å) is predicted to occur between the sidechain of L275 and the quinolone moiety of RCQ06. As shown in Table 1, the experimental data indicate that the determinant of the sensitivity towards RCQ06 is a phenylalanine in position 275, which is naturally present in plasmodial and mammalian cytochrome *b*. Inspection of the model presented in Figure 1C suggests that a favourable aromatic/aromatic interaction may be formed between F275 and the RCQ06 quinolone group, with a larger van der Waals contact area than that observed for the proposed L275/RCQ06 interaction, effectively packing this sidechain against the inhibitor. A similar stabilising aromatic/aromatic interaction, with the aromatic groups oriented perpendicularly, is predicted to form between the sidechain of Y279 and the quinolone moiety of RCQ06. Replacement of Y279 by a serine would destabilise RCQ06, which would explain the observed resistance.

### Conclusions

Acquired resistance to atovaquone developed readily, which is caused by the mutation of the cytochrome *b* residue Y279 (Y268 in *P. falciparum*). New drugs are needed that can circumvent the acquired atovaquone resistance: for example, drugs targeting the Q_i_ site [36], [37] and compounds binding at the Q_o_ site in the haem *b*_l_-proximal region such as azoxystrobin or in the distal site but with different binding interactions than atovaquone [38].

Cytochrome *b* sequences from yeast, human and *P. falciparum* are highly conserved, making yeast an attractive model to investigate sensitivity and resistance to drugs targeting the *bc*_1_ complex. Crystal structures of yeast *bc*_1_ complex are available. Most importantly, genetic tools have been well developed to introduce designed mutations in yeast mitochondrially-encoded cytochrome *b* gene. Yeast is the only organism, with the green algae *Chlamydomonas reinhardtii*, amenable to mitochondrial transformation. By modifying the yeast enzyme, useful tools could be created for the discovery and the analysis of new drugs with potential antimalarial activity and for the study of the development of resistance mutations.

In previous reports, we studied acquired resistance mutations found in parasites and introduced in yeast Q_o_ site [15], [22], [32]. In this study, we generated yeast mutants with modified *Plasmodium*-like Q_o_ sites that mimic the site of the plasmodial enzyme. We tested the sensitivity to atovaquone and azoxystrobin to validate the model. We showed that the differential sensitivity to atovaquone and azoxystrobin, and the high resistance caused by Y279S (Y268S) could be reproduced in the yeast models.

We then used the mutants to study the binding mode of a new molecule RCQ06. The experimental data, together with the model of RCQ06 binding in the Q_o_ site, indicated that the drug shares the same binding pocket than atovaquone. Even though the binding mode of RCQ06 might not be identical to that of atovaquone, the efficiency of binding is affected by the Y279S mutation in the yeast enzyme.

More remodelling of the yeast Q_o_ and Q_i_ sites will be performed to obtain easy-to-use models that mimic more accurately the plasmodial enzyme. These mutants with a *P. falciparum-*like enzyme could be useful tools for the discovery and the analysis of new drugs with potential anti-malarial activity and for the prediction of sensitivity/resistance phenotype associated with naturally occurring cytochrome *b* polymorphisms segregating in parasite populations.
